# Supplementary material for: Impacts of anxiety and socioeconomic factors on mental health in the early phases of the COVID-19 pandemic in the general population in Japan: A web-based survey
Source: PLoS One. 2021 Mar 17;16(3):e0247705. doi: 10.1371/journal.pone.0247705 (PMC7968643; doi:10.1371/journal.pone.0247705)
Supplement: S1 Questionnaire — (DOCX) [file pone.0247705.s001.docx]

**S1 Questionnaire**

**F_Sex: What is your sex?**

1. Male

2. Female

**F_Age_5C: How old are you?**

1. 20-29 years old

2. 30-39 years old

3. 40-49 years old

4. 50-59 years old

5. Over 60 years old

**F_Married: What is your marital status?**

1. Single

2. Married

**F_Job_4C: What is your employment status?**

1. Regular employee

2. Non-regular employee

3. Self-employed and others

4. Not working

**F_Educational background: Which of the following is your final academic background?**

1. Graduated from university/graduate school (natural sciences)

2. Graduated from university/graduate school (social science)

3. Graduated from university/graduate school (humanities)

4. Graduated from university/graduate school (others)

5. Graduated from junior college/technical college

6. Graduated from a vocational school

7. Graduated from high school

8. Graduated from junior high school

9. Others, please specify: ____________

**F_Household income_3C: How much is your annual household disposable income?**

1. ≥6,000K

2. 2,000K-<6,000K

3. <2,000K

**F_Medical history: Q4. Are there any illnesses that you are currently treating for?**

1. Yes

2. No

**F_15: Have you been worried about the following items after the outbreak of the new coronavirus infection?**

|  | Extremely worried | Slightly worried | Neither or not applicable | I am not much worried | No worries |
| --- | --- | --- | --- | --- | --- |
| 1. Vague anxiety without a particular reason. | 1 | 2 | 3 | 4 | 5 |
| 1. Anxiety about the possibility that I get infected. | 1 | 2 | 3 | 4 | 5 |
| 1. Anxiety about the possibility that my family get infected. | 1 | 2 | 3 | 4 | 5 |
| 1. Inability to receive COVID-19 tests immediately. | 1 | 2 | 3 | 4 | 5 |
| 1. Lack of medicine. | 1 | 2 | 3 | 4 | 5 |
| 1. Having trouble in daily life. | 1 | 2 | 3 | 4 | 5 |
| 1. Unavailability of masks. | 1 | 2 | 3 | 4 | 5 |
| 1. Lack of groceries, toilet paper, tissue paper, etc. | 1 | 2 | 3 | 4 | 5 |
| 1. Delays in children’s education | 1 | 2 | 3 | 4 | 5 |
| 1. Impact on financial conditions such as income. | 1 | 2 | 3 | 4 | 5 |
| 1. New work styles, such as telework and remote work. | 1 | 2 | 3 | 4 | 5 |

**Question: From the following, what are you practising to prevent infection?**

**F_Rest: 1) Taking sufficient rest and sleep.**

1. Yes

2. No

**F_Food intake: 2) Having nutritious meals.**

1. Yes

2. No

**F_Exercise: 3) Performing exercises alone, such as marathons and those using DVDs.**

1. Yes

2. No

**F_25_Drink: Please choose one that applies to your drinking habits.**

1. I do not drink at all.

2. I used to drink, but now I stopped.

3. I drink several times in a month.

4. I drink once or twice a week.

5. I drink at least three times or more a week.

6. I drink daily.

**F_26_Smoking: Do you smoke?**

1. I smoke daily.

2. I smoke occasionally.

3. I used to smoke, but now I stopped.

4. I have not smoked before.

**F_K6_2C: The following questions ask about how you have been feeling during the past 30 days. For each question, please circle the number that best describes how often you had this feeling.**

|  | Always | Often | Sometimes | Rarely | Never |
| --- | --- | --- | --- | --- | --- |
| Q1. During the past 30 days, how often did you feel: |  |  |  |  |  |
| 1. nervous? | 1 | 2 | 3 | 4 | 5 |
| 2. hopeless? | 1 | 2 | 3 | 4 | 5 |
| 3. restless or fidgety? | 1 | 2 | 3 | 4 | 5 |
| 4. so depressed that nothing could cheer you up? | 1 | 2 | 3 | 4 | 5 |
| 5. that everything was an effort? | 1 | 2 | 3 | 4 | 5 |
| 6. worthless? | 1 | 2 | 3 | 4 | 5 |
