# Supplementary material for: Impacts of anxiety and socioeconomic factors on mental health in the early phases of the COVID-19 pandemic in the general population in Japan: A web-based survey
Source: PLoS One. 2021 Mar 17;16(3):e0247705. doi: 10.1371/journal.pone.0247705 (PMC7968643; doi:10.1371/journal.pone.0247705)
Supplement: Attachment — Submitted filename: Second.Response_to_Reviewers.docx [file pone.0247705.s004.docx]

**Authors’ response to reviewers:**

**PLOS ONE**

**PONE-D-20-21244**

**Impacts of anxiety and socioeconomic factors on mental health in the early phases of the COVID-19 pandemic in the general population in Japan: a Web-based survey**

**Thank you very much for reviewing our manuscript. We have made the suggested changes as follows:**

**6. Review Comments to the Author

Please use the space provided to explain your answers to the questions above. You may also include additional comments for the author, including concerns about dual publication, research ethics, or publication ethics. (Please upload your review as an attachment if it exceeds 20,000 characters)**

**Reviewer #1: Thank you for your polite reply.

1. #1: The authors asked the medical history of the participants and noted whether the participant visits the hospital regularly. Could you tell me the department in the field of medical care?
(This may affect the results.)

Response:
Thank you for this question. Unfortunately, we did not ask which department the participants regularly visit in this study.

＝＞ Thank you for your polite answer.**

**Response:**

**Thank you for your time to review our manuscript.**

**2. #2: Would you tell me more data of the method of sampling?
The authors should follow the Checklist for Reporting Results of Internet E-Surveys (CHERRIES) to minimize the potential bias.

Response: Thank you for your recommendation. We followed the CHERRIES and added detailed information to the Methods section as much as possible. Please refer to the Methods section.

=> Thank you for your polite reply.**

**Response:**

**Thank you for your comment.**

**Reviewer #2: The author has properly amended the description of the original manuscript in almost all areas with argument.**

**However, I never understand why the author claims that "people demand receiving the PCR test as a human right as a Japanese citizen."**

**To begin with, the author themselves wrote they don't believe PCR test should be provided to everyone who want to do immediately in the former section of the response letter. Their comments are quite paradoxical.**

**I am doubtful that the author understand the sensibility and specificity of an examination. The current sensitivity of PCR is estimated as 70%. Regarding specificity, there is controversy, estimated between 99% to 99.99%. Even if the specificity is 99.999%, ten people of a million without COVID-19 will receive false positive, leading to unnecessary seclusion and wasting medical resource. False negative case can be more serious. Discussing providing PCR alone is quite risky and unrealistic.
Again, I have to say that the outcome of the present survey does not support the idea that allowing any people to undertake the PCR test is appropriate. It is an issue of public health, not human right. Human right is an essential matter which should never been violated. It is far from protecting human right to justify things the majority wants to do. If the vast majority of Japanese support racism, should the Japanese government take such a policy? Ridiculous. As far as the author use the term "human right" in an arbitrary manner, the author's manuscript does not deserve to be read.**

**Response:**

**Thank you for your time to review our manuscript.**

**Please accept our sincere apology regarding improper terminology usage and taking up your crucial time.**

**First, we sincerely apologize for using the word “human rights” in an unthoughtful manner. If you allow us, we would like to remove the word and the corresponding sentence, mentioned in our previous response, Comment 24, to reviewers.**

**Second, we deleted the word “PCR tests.” Our original question was “F_15: Have you been worried about the following items after the outbreak of the new coronavirus infection?” The sub-question we provided to the respondents was “3) Inability to receive COVID-19 tests immediately.” In the early stages of the pandemic in Japan, “COVID-19 tests” meant “PCR tests”; however, writing “PCR tests” is not accurate, and is different from the original question. Thus, we deleted the word “PCR tests” and rewrote as “COVID-19 tests” in the manuscript. In addition, we also rewrote “at will” to “immediately,” in an attempt to describe the questions more accurately.**

**Third, we realized that people’s circumstances at the time this survey was conducted (March) were different from when this manuscript was written (November). Therefore, we added more information on the differences between the testing systems in place during March and November 2020 as follows:**

**“****At the early stages of the pandemic, the COVID-19 testing system was fragile. Patients were unable to receive COVID-19 tests immediately, even when physicians determined that it was necessary. The result of this study may reflect the poor testing system during the study period—March 2020. However, the system has improved day by day; the numbers of COVID-19 tests taken increased significantly from 13,026 cases in March 15 to 3,035,324 cases in November 15, 2020 (47, 48). Moreover, a variety of tests are now available.”**

**“Providing accurate reasons and information about COVID-19 testing to people who cannot undergo COVID-19 testing may help reducing the level of anxiety.”**
