# Supplementary material for: Impacts of anxiety and socioeconomic factors on mental health in the early phases of the COVID-19 pandemic in the general population in Japan: A web-based survey
Source: PLoS One. 2021 Mar 17;16(3):e0247705. doi: 10.1371/journal.pone.0247705 (PMC7968643; doi:10.1371/journal.pone.0247705)
Supplement: Attachment — Submitted filename: Response_to_Reviewers.docx [file pone.0247705.s005.docx]

**Response to reviewers:**

**PLOS ONE**

**PONE-D-20-21244**

**Impacts of anxiety and socioeconomic factors on mental health in the early phases of the COVID-19 pandemic in the general population in Japan: a Web-based survey**

**Reviewers' comments:**

**Reviewer's Responses to Questions**

**Comments to the Author**

**1. If the authors have adequately addressed your comments raised in a previous round of review and you feel that this manuscript is now acceptable for publication, you may indicate that here to bypass the “Comments to the Author” section, enter your conflict of interest statement in the “Confidential to Editor” section, and submit your "Accept" recommendation.**

**Reviewer #3: (No Response)**

**2. Is the manuscript technically sound, and do the data support the conclusions?**

**The manuscript must describe a technically sound piece of scientific research with data that supports the conclusions. Experiments must have been conducted rigorously, with appropriate controls, replication, and sample sizes. The conclusions must be drawn appropriately based on the data presented.**

**Reviewer #3: Yes**

**3. Has the statistical analysis been performed appropriately and rigorously?**

**Reviewer #3: (No Response)**

**4. Have the authors made all data underlying the findings in their manuscript fully available?**

**The PLOS Data policy requires authors to make all data underlying the findings described in their manuscript fully available without restriction, with rare exception (please refer to the Data Availability Statement in the manuscript PDF file). The data should be provided as part of the manuscript or its supporting information, or deposited to a public repository. For example, in addition to summary statistics, the data points behind means, medians and variance measures should be available. If there are restrictions on publicly sharing data—e.g. participant privacy or use of data from a third party—those must be specified.**

**Reviewer #3: No**

**5. Is the manuscript presented in an intelligible fashion and written in standard English?**

**PLOS ONE does not copyedit accepted manuscripts, so the language in submitted articles must be clear, correct, and unambiguous. Any typographical or grammatical errors should be corrected at revision, so please note any specific errors here.**

**Reviewer #3: Yes**

**6. Review Comments to the Author**

**Please use the space provided to explain your answers to the questions above. You may also include additional comments for the author, including concerns about dual publication, research ethics, or publication ethics. (Please upload your review as an attachment if it exceeds 20,000 characters)**

**Reviewer #3: Thank you for giving me a chance to review this manuscript. I confirmed the authors have already amended the questions from the other reviewers in the previous rounds. The paper now is well written and has scientific meaning. Therefore, I suggest its acceptance after fixing several points below.**

**Response:**

**Thank you very much for reviewing our manuscript. We have made the suggested changes.**

**Major:**

**1. I can understand that the authors changed the expressions of “PCR tests” to “COVID-19 tests” to answer previous reviewer 2’s question. However, I think it is not acceptable to change the questionnaire after it has been answered. Because if we ask a question in another way, the response may be different. Authors may add some explanation about “PCR tests here were the only tests for COVID-19 at that time.” However, I do not think it is acceptable to change the original question.**

**Response:**

**Thank you for your comment.**

**We totally agree with your comment. We have never changed the expression and meaning of the original questions. We are afraid that it is misunderstanding due to our inaccurate and confusing reply to the inquiry from the editor, that is “We have revised the questionnaire as part of the second review process.” Obviously, it was not correct. The truth is that our translation from Japanese statement in the original questionnaire to English explanation in our manuscript, replies to the second reviewer, and questionnaire in the supplementary data were wrong. Please understand what we revised was not the original questionnaire but only an English translation.**

**Specifically, we found a careless mistake of the translation in the question No. F15-4 (the original question in Japanese language “検査がすぐに受けられないこと”): The first author translated it as “Not being able to take the PCR test immediately”. However, during review process, the co-authors noticed that the translation was incorrect as the word “the PCR test” is not accurate and inconsistent with the original question in Japanese. Thus, we revised the translation as “Inability to receive COVID-19 tests immediately” in the manuscript and replies to the second reviewer as well as the English-translated questionnaire in the supplementary data.**

**We sincerely apologize for the confusion we have caused.**

**2. Supplemental data for all the response data were not included in this revision, while PLOS ONE asked authors to upload their data somewhere. I found the data in revision R1. I did not know why authors deleted them.**

**Response:**

**Thank you very much for your comment. We apologize for not knowing that we had to submit the data set in every revision. As you mentioned that we submitted the data set for the first time, we thought that it was not necessary to submit it again. However, we would like to resubmit the data set including the revised translation of the questionnaire with this revised manuscript.**

**Minor points:**

**3. P.17 L.229 Regular employees among females (AOR 1.125 …)**

**While in Table 3 (P.20), it is “Non regular” employee (AOR “1.215”). Please confirm both the label and the number, and fix either one.**

**Response:**

**Thank you for pointing out the mistake. The number in Table 3 is correct. The number of the manuscript (AOR 1.125) has been revised.**

**4. P.17 L.230-232 there are typos. Shouldn't all “≥ 6 million” be “< 6 million?” (according to Table 3.)**

**Response:**

**Thank you for pointing out the mistake. Three parts of the manuscript have been corrected.**

**5. P.20 Table 3 Some names of rows were difficult to be understood and different from the questionnaire, e.g., “What I may be infected with the virus,” “What may affect my family.”**

**Response:**

**Thank you for your comment.**

**We apologize for the incomprehensibleness and the differences between the questionnaire and the names of the rows in Table 3. We renamed the names of the rows in Table 3 and also Table 1, so that the names become consistent with the translated questionnaire. We also revised several wordings so that they become consistent among the manuscript, tables, and the translated questionnaire.**

**6. P.22 L.326 “Three previous studies in China and Turkey also reported that Chinese female …”**

**The study from Turkey (ref. 6) should not report Chinese results. Therefore, please remove “Chinese” here.**

**Response:**

**Thank you for your suggestion. We removed the word “Chinese”.**

**7. P. 22-23 “Previous research concluded that females were at a higher risk of mental health problems than men (31).”**

**Ref. 31 is a paper on post-injury mental health problems. It seems to be a particular case and a little far from the current results. Could the authors revise this part?**

**Also, in the following sentence, “these three previous studies” is written. It is better to remove “three,” if the authors mention both Ref 15 and 31, together with Ref 3, 6, and 30.**

**Response:**

**Thank you for your suggestion. We deleted the line mentioned above and the word “three”.**

**8. P. 28 L.430 “using a PC device” via the internet.**

**To my knowledge, such kind of survey can also be answered by smartphones. If so, please remove using a PC device or add some other means to make it clear.**

**Response:**

**Thank you for your suggestion. We removed the word “using a PC device” and added a sentence “a device which can connect to the Internet”.**
