# Supplementary material for: Impacts of anxiety and socioeconomic factors on mental health in the early phases of the COVID-19 pandemic in the general population in Japan: A web-based survey
Source: PLoS One. 2021 Mar 17;16(3):e0247705. doi: 10.1371/journal.pone.0247705 (PMC7968643; doi:10.1371/journal.pone.0247705)
Supplement: Attachment — Submitted filename: Response_to_Reviewers.docx [file pone.0247705.s006.docx]

**Response to reviewers:**

**PLOS ONE**

**PONE-D-20-21244**

**Impacts of anxiety and socioeconomic factors on mental health in the early phases of the COVID-19 pandemic in the general population in Japan: a Web-based survey**

**6. Review Comments to the Author

Reviewer #3: Dear editor and authors,

Thank you for giving me a chance to reconfirm this manuscript: “Impacts of anxiety and socioeconomic factors on mental health in the early phases of the COVID-19 pandemic in the general population in Japan: A web-based survey.” It is very interesting and meaningful to understand the citizens’ mental health in the early stage of the COVID-19 pandemic. The data are sampled per the Japanese population and have a sufficient number of respondents to give the results and conclusion.

I have reconfirmed the revision of this manuscript, and most of the questions have been answered. Although two minor points are listed below, I think they can be fixed during the publication steps. Therefore, now, I advise the publication of this manuscript in PLOS ONE.

Response:**

**We appreciate the reviewer to review our manuscript and give us productive comments. For the minor points suggested below, we modified the manuscript according to the reviewer’s comments.**

**Minor points:
1. P. 17 L. 229 “such as regular employees among females…”
Shouldn’t it be non-regular employees? According to Table 3, regular employees are the reference. The odds ratio is non-regular employees/regular employees.**

**Response:**

**We added the word “non-” and the sentence is as follows:**

**“as non-regular employees among females (AOR1.215 [95% CI: 1.025–1.440])”**

**2. P. 8 L.136 “Have you been worried the following items …”
“about” should be added after “worried” for English grammar.**

**Response:**

**Thank you for your suggestion. We added the word “about” and the sentence is as follows:**

**“Have you been worried about the following items after the outbreak of the new coronavirus infection?”**
